# When spinal instrumentation revision is not an option: Salvage vertebral augmentation with polymethylmethacrylate for mechanical complications: A systematic review^☆^

**DOI:** 10.1016/j.bas.2023.101726

**Published:** 2023-03-01

**Authors:** Derek T. Cawley, Kiran Divani, Roozbeh Shafafy, Aiden Devitt, Sean Molloy

**Affiliations:** a Mater Private Hospital, Dublin, 1, Ireland; b Royal London Hospital, Barts Health, London, UK; c Dept of Spinal Surgery, RNOH Stanmore, Brockley Hill, Stanmore, HA7 4LP, UK; d Dept of Spinal Surgery, University of Galway, Ireland

**Keywords:** Pedicle screws, Polymethylmethacrylate, Vertebroplasty, Non-union, Junctional failure, Screw loosening, Pseudarthrosis

## Abstract

**Intoduction:**

Mechanical complications from spinal fusion including implant loosening or junctional failure result in poor outcomes, particularly in osteoporotic patients. While the use of percutaneous vertebral augmentation with polymethylmethacrylate (PMMA) has been studied for augmentation of junctional levels to offset against kyphosis and failure, its deployment around existing loose screws or in failing surrounding bone as a salvage percutaneous procedure has been described in small case series and merits review.

**Research Question:**

How effective and safe is the use of PMMA as a salvage procedure for mechanical complications in failed spinal fusion?.

**Materials and Methods:**

Systematic search of online databases for clinical studies using this technique.

**Results:**

11 studies were identified, only consisting of two case reports and nine case series. Consistent improvements were observed in pre- to post-operative VAS and with sustained improvements at final follow-up. The extra- or para-pedicular approach was the most frequent access trajectory. Most studies cited difficulties with visibility on fluoroscopy, using navigation or oblique views as a solution for this.

**Discussion and Conclusions:**

Percutaneous cementation at a failing screw-bone interface stabilises further micromotion with reductions in back pain. This rarely used technique is manifested by a low but increasing number of reported cases. The technique warrants further evaluation and is best performed within a multidisciplinary setting at a specialist centre. Notwithstanding that underlying pathology may not be addressed, awareness of this technique may allow an effective and safe salvage solution with minimal morbidity for older sicker patients.

## Highlights

• Percutaneous cementation at a failing screw-bone interface arrests further micromotion with pain reductions.
• This rarely used technique is manifested by a low but an increasing number of reported cases.
• It warrants further evaluation but may allow an effective and safe salvage solution for older sicker patients.

## 1 Introduction

Over 30% of adult spinal deformity operations require re-operation within 5 years, with instrument failure cited as the most common cause followed by pseudarthrosis, junctional failure and adjacent segment disease (Puvanesarajah et al., 2016). Loosening of osteosynthesis instrumentation occurs in up to 27%, especially in osteoporotic and for revision cases (Okuyama et al., 2001; Diebo et al., 2015). Senior citizens constitute an expanding surgical demographic, displaying bone qualities that pre-dispose to among other outcomes, mechanical complications of spinal instrumentation. Ensuing cyclic caudocephalad screw toggling causes localized cavitation and loosening. The structural failure that occurs with PJF can present as vertebral body fracture, implant pull-out or breakage causes junctional end-vertebral collapse.

The use of polymethylmethacrylate (PMMA) has expanded since its introduction by Galibert and Deramond in 1984 to include kyphoplasty and fenestrated pedicle screws with cement augmentation (Galibert et al., 1987). Percutaneous vertebral augmentation with PMMA in deficient bone around a failing screw-bone interface as a salvage procedure has been described in biomechanical testing as leading to similar or higher fatigue strengths compared with those of the initially augmented screws (Weiser et al., 2021). More recently, this technique has been described as a salvage procedure in isolated clinical cases and small series. The rational for a systematic review is to identify and assess research papers on this topic and in particular, to identify the participants, interventions, comparisons and outcomes (PICO).

## 2 Methods

### 2.1 Search strategy and study selection

A systematic search of databases including Pubmed, MEDLINE, EMBASE and Cochrane library, Web of Science and OVID. Languages included English and French. The search words included: cement augmentation polymethylmethacrylate, vertebroplasty, pedicle screw loosening, instrumentation. A further search was conducted of papers citing or cited by relevant papers. Using the PRISMA guidelines (Moher et al., 2009), we searched for studies published up to July 2022 that evaluated salvage percutaneous polymethylmethacrylate augmentation for pedicle screw loosening. Two review authors independently selected studies, assessed risk of bias and extracted data, with input from the third author. Assessment of case reports and case series was guided by the CARE checklist (Murad et al., 2018).

### 2.2 Inclusion and exclusion criteria

Studies were included in the analysis if the following inclusion criteria were met: (1) any clinical study design (2) study population: participants were patients, over 18 years of age with failed spinal fusion from a loosened pedicle screw; (3) who were treated with a salvage percutaneous cement augmentation at the level of the loosened pedicle screw(s); (4) clinical outcomes: pre- and post-operative visual analogue score (VAS) or patient global impression of change (PGIC). Studies that did not meet the above criteria were excluded from selection. Exclusion criteria were patients with pedicle screw loosening due to infection, patients who were treated with cement at the time of the original screw insertion, patients who underwent open salvage surgery or where the loose pedicle screw was removed and revised to a cemented screw.

### 2.3 Data extraction

Data were extracted independently by two authors, without blinding to the title and author affiliation. Relevant information was extracted from studies, included: (1) the title; (2) authors; (3) year of publication; (4) sample size; (5) type of intervention; (6) surgical approach; (7) duration of the follow-up; (8) clinical outcomes as reflected in the pre-, post- and follow-up VAS scores (9) cement dosage; (10) peri-operative complications and (11) bone cement leakage. We documented whether industry funding was received based on disclosures.

### 2.4 Results

**Fig. 1:**
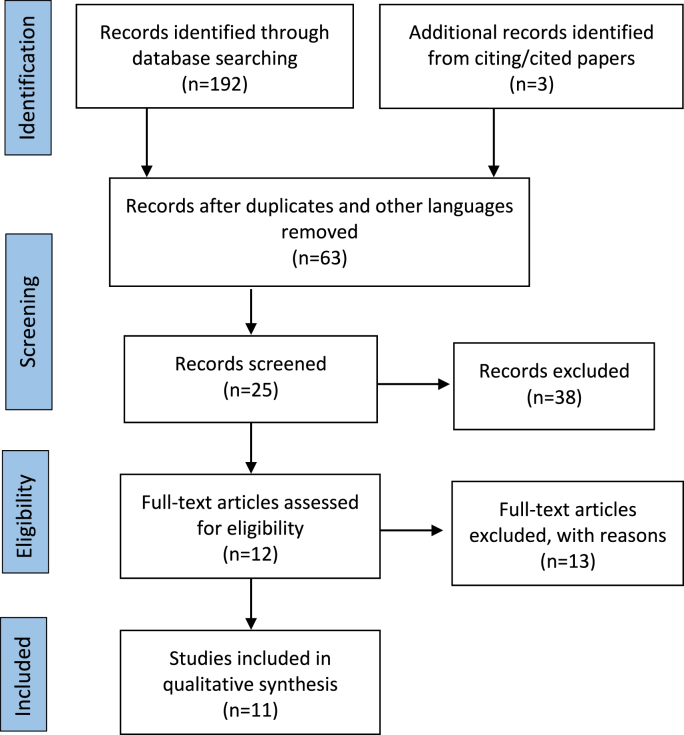
PRISMA flow diagram for selection of studies based on inclusion criteria during systematic review (Moher et al., 2009).

A total of 195 citations or papers were identified (Fig. 1). 131 citations were excluded on the basis of irrelevance despite containing the appropriate search words or were in another language. 38 studies were excluded for an inappropriate intervention (e.g. cement augmented screw insertion). 25 were identified during the search and full text copies of all potentially relevant studies were obtained. 13 demonstrated evidence of additional surgical measures (e.g. open screw revision) Finally, 11 studies were specific to the research question: two case reports and nine case series (Table 1).

**Table 1:** Summary of findings.

Study, Year	Design	No. Of patients	Initial Surgery to Cement Period	Pre-op to Post-op VAS/PGIC	Adverse Events	Subsequent Revision
Divani, 2018 (Divani et al., 2018)	Case Report	1	N A	9/10–1/10	No	No
Amoretti, 2014 (Amoretti et al., 2015)∗	Case Series	4	24 months	9/10–0/10	1 psoas hematoma	No
Clerk-Lamalice, 2018 (Clerk-Lamalice et al., 2018)	Case Report	1	5 weeks	9/10–2/10	No	No
Fu, 2018 (Fu and Li, 2018)	Case Series	10	N A	5.9–2.5	No	2/10, incidental trauma & osteoporosis
Xu, 2014 (Xu et al., 2014) ∗	Case Series	11	5 months	8.4–1.5	No	1/11, 5 months, unilateral rod fracture revised
Yilmaz, 2006 (Yılmaz et al., 2022)	Case Series	15	2 months	7.5–2.4/10	No	No
Yun (Yun et al., 2016)	Case Series	8	NA	7.2–5.6	Cement leakage in 2,	Cage change in 2, infection in 1
Cianfoni (Cianfoni et al., 2018)	Case Series	29	14 months	87% PGIC 5-7	1 access failure	5 required revision- infection, PJK, persistent loosening
Do (Do et al., 2020)	Case Series	7	1–8 months	NA (radiology only)	No	2 required revision
Prestat (Prestat et al., 2021)∗∗	Case Series	4	<24 months	8/10–0/10	1 small intramuscular hematoma	No
Wang∗∗∗ (Wang et al., 2021)	Case Series	32	2–6 years	7.97–2.34	2 cement leaks without consequence	5 had removal of screws

N A: Not available, ∗All metastatic tumor patients, ∗∗ All trauma patients, ∗∗∗Solid fusion with loose pedicle screws.

### 2.5 Data analysis

The selection process of relevant studies is summarized in Fig. 1. The series was too small for a metanalysis but collated data from all papers demonstrate a mean age of 70 years (38–80 years). The series included patients from China, France, Italy, Korea, Taiwan, Turkey, United Kingdom and United States (Divani et al., 2018; Amoretti et al., 2015; Clerk-Lamalice et al., 2018; Fu and Li, 2018; Xu et al., 2014; Yılmaz et al., 2022; Yun et al., 2016; Cianfoni et al., 2018; Do et al., 2020; Prestat et al., 2021; Wang et al., 2021). Relevant investigations included plain radiography and CT (Fu and Li, 2018; Yılmaz et al., 2022; Cianfoni et al., 2018; Do et al., 2020; Prestat et al., 2021; Wang et al., 2021), MRI and CT SPECT (Divani et al., 2018; Amoretti et al., 2015), CT and MRI (Xu et al., 2014) and additional dynamic radiographs (Fu and Li, 2018). The pathology which accompanied the diagnosis of pedicle screw loosening was proximal junctional failure (PJF) with a long construct (Divani et al., 2018; Clerk-Lamalice et al., 2018; Yun et al., 2016; Do et al., 2020), instrumented fusion for trauma (Amoretti et al., 2015; Prestat et al., 2021), degenerative (Fu and Li, 2018; Yılmaz et al., 2022), tumor (Xu et al., 2014) or a combination (Cianfoni et al., 2018). The operated levels from 3 series included T11- S1 (Fu and Li, 2018; Yılmaz et al., 2022; Wang et al., 2021). There was no patient lost to follow up. In one series, cases had established solid fusion on plain radiography and CT, but with persistent pain requiring PMMA (Wang et al., 2021).

### 2.6 Assessment of quality of evidence

The overall assessment of the evidence displays a collection of low certainty evidence (Table 2). There are no details on co-interventions (e.g. analgesia; physiotherapy) and studies did not report on blinding or independent outcome assessment. The levels of performance and detection bias were potentially high. No study had a comparison group, including conservative or surgical treatments. Two studies received funding from a company responsible for manufacture of kyphoplasty equipment (Xu et al., 2014) and spinal equipment (Cianfoni et al., 2018). However, all but one included studies had consecutive VAS or PGIC scores and some also reported EQ-5D (EuroQol 5D), ODI (Oswestry Disability Index) and SRS22 (Scoliosis Research Society) outcomes (Divani et al., 2018), Japanese Orthopedic Association (JOA) scores (Yılmaz et al., 2022), Low Back Outcome Score (LBOS) (Prestat et al., 2021) or Roland Morris (RMDQ) (Wang et al., 2021).

**Table 2:** Assessment of quality of evidence (Moher et al., 2009).

Study, Year	Selection	Ascertainment	Follow up	Reporting
Divani, 2018 (Murad et al., 2018)	Very good- Retrospective, detailed description of indications	Very good- MRI, DEXA and CT SPECT	12 months	Reporter bias, VAS, ODI, EQ5D
Amoretti, 2014 (Divani et al., 2018)∗	Good- Prospective, no exclusion criteria, comorbidity was the inclusion criterion	Very good- Scintigraphy, MDT, CT	6 months	Reporter bias, radiological & clinical outcome only included VAS
Clerk-Lamalice, 2018 (Amoretti et al., 2015)	Poor- Not described	Good- CT	3 months	Reporter bias, VAS, function & ROM reported
Fu, 2018 (Clerk-Lamalice et al., 2018)	Good- Retrospective, chart review	Good- Radiograph and CT	14 months	VAS & radiological outcomes
Xu, 2014 (Fu and Li, 2018) ∗∗	Good- Retrospective, clear inc/exc criteria	Good- MR, CT	24 months	VAS, Functional Mobility & Analgesic Scale
Yilmaz, 2006 (Xu et al., 2014)	Good- Retrospective	Good- Radiograph and CT	8 months	VAS & radiological outcomes
Yun (Yılmaz et al., 2022)	Good- Retrospective	Unclear	12 months	VAS, ODI
Cianfoni (Yun et al., 2016)	Good- Retrospective	Good- Radiograph and CT	16 months	PGIC, clinical & revision rates only
Do (Cianfoni et al., 2018)	Good- Retrospective	Good- Radiograph	7–20 months	Radiographic, clinical & revision rates only
		Excellent		
Prestat (Do et al., 2020)	Good- Retrospective	Good- Radiograph and CT	12 months	VAS, LBOS & radiological outcomes
Wang (Wang et al., 2021)	Good- Prospective	Radiograph and CT	12 months	VAS, RMQD

### 2.7 Techniques

Cases performed by a radiologist were more likely to be CT and fluoroscopy guided (Amoretti et al., 2015; Xu et al., 2014; Prestat et al., 2021) or with intra-operative navigation if done by a spinal deformity surgeon (Divani et al., 2018). However, navigation guided surgery, usually mandates attaching a reference frame to bony anatomy which implies making a separate incision for this purpose. As a technical point, impaction may distort navigation accuracy, thus insertion of any trocar or Jamshidi needle, if using navigation must be inserted without movement of the spine relative to the reference frame. CT-guided procedures, at least, provide a real-time image of the position of the needle. Some operators performed the procedures under local anesthetic (Clerk-Lamalice et al., 2018; Fu and Li, 2018; Cianfoni et al., 2018; Do et al., 2020). All cases were positioned prone, thus maximizing the space for cement to be injected.

A latero- or extra-pedicular lumbar approach was taken along the path of the loosened screw, or parallel route to a sacral screw (Table 3). In the case of iliac screw loosening, a lateral approach was taken (Amoretti et al., 2015; Prestat et al., 2021). Application of cement was started from the distal apex of the screws and filled backwards (Amoretti et al., 2015; Fu and Li, 2018; Yılmaz et al., 2022; Prestat et al., 2021). The anterior vertebral cortex was probed to ensure that the tip of the trocar remained inside the vertebral body (Clerk-Lamalice et al., 2018). An extra-pedicular route to the vertebral body is preferable to a parallel transpedicular trocar as the existing screw and rod may block trocar access and obscure the radiographic view (Fu and Li, 2018; Wang et al., 2021). Yilmaz et al. advocated accessing through Kambin's triangle (Yılmaz et al., 2022). This may also facilitate cement augmentation to both sides of the vertebra from one access point (Xu et al., 2014; Yılmaz et al., 2022). Two studies preferred trans-pedicular approach for the lumbar and lower thoracic spine and extra-pedicular for the mid and upper spine (Cianfoni et al., 2018; Do et al., 2020). The oblique view can help to visualize the screw base so that potential for posterior cement extrusion can be monitored (Fu and Li, 2018). A curved needle was helpful to direct the cement into different regions of the vertebral body if the screws limited the position of the introducer needle (Xu et al., 2014). For PJF, the vertebral endplates may be pierced to allow a continuum of cement flowing through the upper instrumented vertebral level proximally into the disc and into the cephalad vertebral body, through the use of three cannulae (Divani et al., 2018; Do et al., 2020).

**Table 3:** Peri-operative details.

Study	Type of Anesthetic	Imaging Format	Approach	Final Trocar Position
Divani (Amoretti et al., 2015)	General	Navigation	Superior to screw	Anterior body, suprajacent disc and suprajacent vertebra
Amoretti (Amoretti et al., 2015)	Local	CT and Fluoro	Latero-pedicular	Tip to tip, then retracted filling the vertebra
Clerk-Lamalice (Clerk-Lamalice et al., 2018)	Sedation & Local	Fluoro	Para-pedicular	Anteromedial to screw, filling the anterior 2/3 of the vertebral body
Fu (Fu and Li, 2018)	Local	Fluoro	Latero-pedicular	Inside the anterior vertebral cortex, extended towards posterior aspect
Xu (Xu et al., 2014)	NA	CT and Fluoro	Sup- or Lateral extra-pedicular	Curved needle, advanced coaxially, allowing access to both the contralateral and unilateral side
Yilmaz (Yılmaz et al., 2022)	General	Fluoro	Kambin's triangle	Vertebral body- loose zone or fracture cavity
Yun (Yun et al., 2016)	NA	Fluoro	Para- & trans-pedicular	NA
Cianfoni (Cianfoni et al., 2018)	Sedation & Local	Fluoro	Para- & trans-pedicular	Flexible beveled trocar, position adjusted depending on location of osteolysis
Do (Do et al., 2020)	Local	Fluoro	Para- & trans-pedicular	Site of fracture
Prestat (Prestat et al., 2021)	Local	CT and Fluoro	Lateral -pedicular	Distal tip of screw
Wang (Wang et al., 2021)	NA	Pre-op CT and Fluoro	Lateral -pedicular	Junction of pedicle and vertebral body

PMMA was used in all cases and volume varied from 3 ​mls (Amoretti et al., 2015; Yılmaz et al., 2022) to 12 ​mls per level (Do et al., 2020).

None of the patients experienced vertebral cement augmentation-related complications such as neural element compression or cement embolization. Consistency of the cement was reported as low viscosity so as to allow flow around the screw (Prestat et al., 2021) to high viscosity (Divani et al., 2018; Cianfoni et al., 2018).

### 2.8 Peri-operative Outcomes (Table 2)

Length of stay varied from 1.2 days (Fu and Li, 2018) to 7 days (Yılmaz et al., 2022). Patients were mobilized on the first day post-operatively. The extra-pedicular and lateral parallel approaches each caused one psoas hematoma which self-resolved over 7 days (Amoretti et al., 2015; Prestat et al., 2021).

### 2.9 Radiological outcomes

One of the PJF patients demonstrated mild kyphosis correction on prone positioning which was sustained at 1 year (Divani et al., 2018). CT at one month demonstrated no further loosening or collapse in the trauma patients (Amoretti et al., 2015). Otherwise plain radiography was used to assess outcomes. In cases of kyphotic fracture, this was not significantly less kyphotic at one year (Do et al., 2020).

### 2.10 Clinical outcomes

Patients demonstrated decreases in VAS from pre-operatively to post-operatively (Table 1, VAS (2–3/10 on post-operative day 1) (Amoretti et al., 2015; Fu and Li, 2018; Yılmaz et al., 2022; Prestat et al., 2021)). Post-operative VAS was reflective of a similar VAS at 1 year. Cianfoni et al. (2018) reported PGIC of 7 (extremely better) in 30% and of 6 in 30% (much better). Yun et al. (2016) reported poorer VAS scores than other series (pre-post 7.2–5.7) but this series included significantly more patients with loosening around corpectomy cages reflecting a more morbid cohort. Wang et al. (2021) reported RMDQ of 16.75 ​± ​1.84 reducing to 7.21 ​± ​4.08 and significantly correlated with pain reduction.

Fu et al. described two revision surgery cases (Fu and Li, 2018). One post-fall 4 months after his L2 percutaneous augmentation suffering two broken rods and kyphosis which were revised from to T11-S1. Notably, the cement-augmented pedicle screws remained stable within L2. The second patient, an 81year-old who suffered PJK and was revised to a T10-L5 fusion. One tumor patient warranted a kyphoplasty for a compression fracture at L1, the previously decompressed tumor site (Xu et al., 2014). None of the studies suggested that there were complications or difficulties during open revision surgery as a result of the prior percutaneous salvage procedure.

### 2.11 Experience of the authors

With adoption of the above research, the authors have used these techniques for salvage cases and have additional technical comments. Pre-operative CT analysis is crucial for planning. Guidance of the trocar is significantly more difficult than with vertebroplasty and as docking the trocar on a screw is difficult, a cross hairs (“bull's eye”, orthogonal to image intensifier) approach, as opposed to a triangulation approach is best for accurate trocar placement. The image intensifier will require oblique positioning, so that the projection of the rod is medial to the screw. Given the oblique trajectory, a long trocar (15–20 ​cm) may be required. A beveled trocar (as opposed to diamond head) will facilitate oblique docking where the bevel is facing away to stop sliding. Ensure that the bevel fully is inside the bone (if visibility allows).

If using a navigated or robotic system, it is crucial to ensure that the port diameter is not significantly larger than the trocar so as to reduce wander and leakage. A highly viscous cement is recommended, bearing in mind that under high insertion pressure, it will speed up the cure time. It is important to recognize tactile feedback from the cement and in particular to stop if there is a loss of resistance. Repeated lateral-projection fluoroscopy is needed with careful monitoring for back flow that may indicate intra-canal or intra-foraminal leakage. It is also worth combining cementation of loose screws with intra-discal cement placement (if space allows), - cement discoplasty as previously reported, to confer additional stability and anterior column loading.

## 3 Discussion

Percutaneous cement augmentation procedures have recently shown significant benefit in patients with degenerative spinal instability and in the presence of a vacuum disc phenomenon (Varga et al., 2015; Kiss et al., 2019). This systematic review identified that this little-known technique demonstrated significant reductions in pain after percutaneous cement augmentation at a failing screw-bone interface. While studies were of heterogeneous cases, including trauma, degenerative, tumor and deformity cases, they all included loose instrumentation, which when stabilized displayed reliable improvements in pain from pre-operative to post-operative status. Consistently, post-operative VAS was similar to VAS at final follow-up. The cement arrests any further micromotion providing immediate, excellent mechanical stability at the failing segment. The extra- or para-pedicular approach was the most frequent access trajectory and the cement volume was mostly 3 ​mls per level. Most studies cited difficulties with visibility on fluoroscopy, citing CT-guidance or oblique views as a solution for this.

**Fig. 2:**
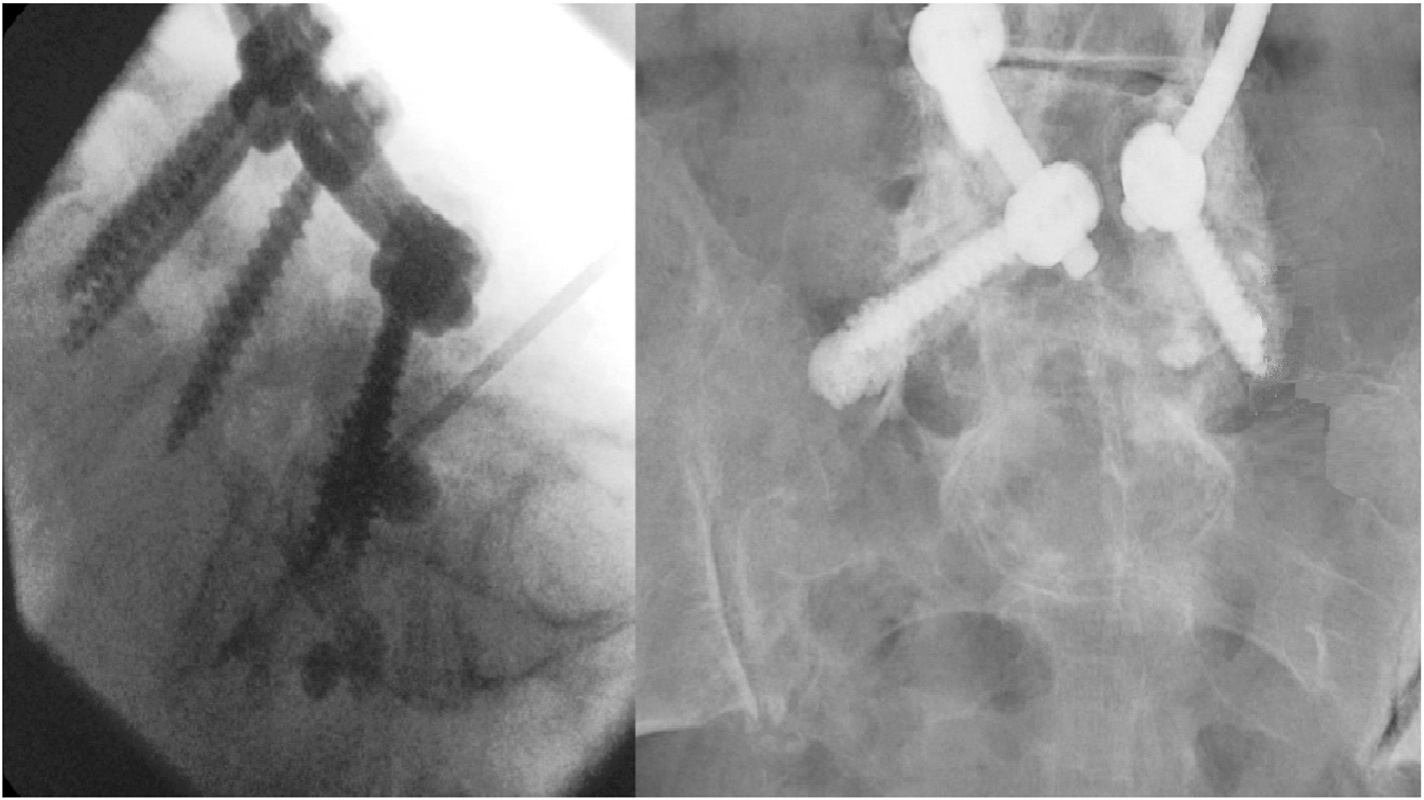
a & 2b: Lateral intra-operative fluoroscopy and postero-anterior post-operative radiography of salvage vertebral augmentation with PMMA around loose L5 screws (sacralized L5, previously revised with divergent trajectory, screw loosening post revision). Anterior cement leakage noted through anterior wall defect, no associated symptoms. Vacuum Disc Phenomenon noted at disc space indicative of persistent mobility.
• Segmental stress-strain mismatch at the end of a long construct, with loss of vertebral integrity, usually PJF (Fig. 3a and b).

Osteoporosis is invariably present in these patients, thus medical treatments and appropriate rehabilitation should incorporate this. Two causative pathologies were evident from this series:

• Localized stress-strain mismatch with persistent motion, screw loosening, localized bone resorption and failure of fusion (Fig. 2a and b).

**Fig. 3:**
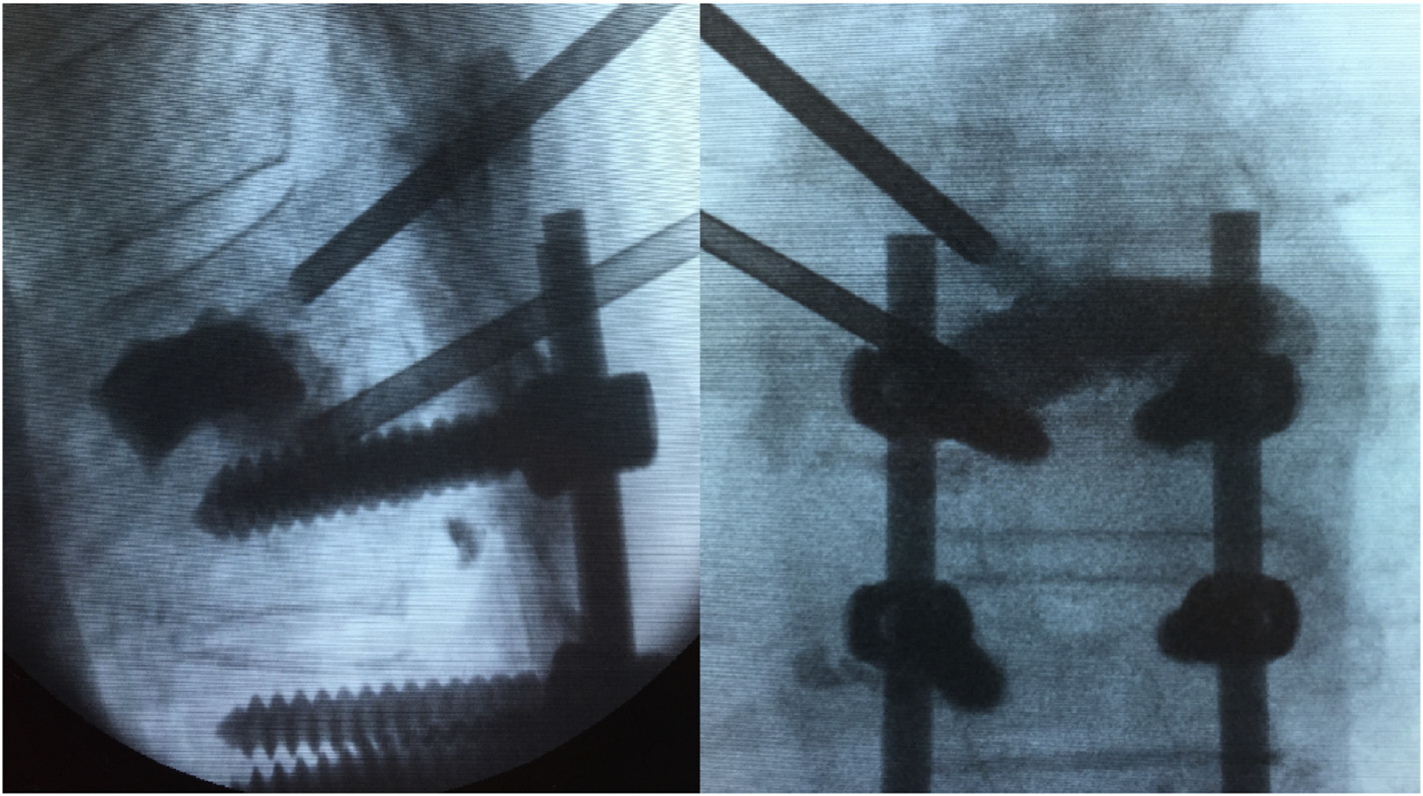
a & 3b: Lateral and postero-anterior intra-operative fluoroscopy views of treatment of loosened pedicle screws. In this case, the accompanying proximal junctional failure prompted the option of vertebroplasty at the adjacent level.

Optimizing treatments thus rely on identifying causative pathologies requiring localized backfilling of the screw track in the former and approximating vertebroplasty in the latter (Fig. 2, Fig. 3 respectively).

Proximal junctional failure (PJF) is a common complication of adult degenerative deformity and extension of instrumentation may be otherwise inevitable, all too frequently failing on a patient's expectations. Thus, PMMA insertion at the upper instrumented level (UIV) and UIV level-plus one, may be considered given the high morbidity alternatives (Fig. 3a and b). Early PJF with construct failure however, has more underlying alignment mismatch characteristics and it seems unlikely to be salvaged by PMMA reinforcement at the most cephalad level. While prone positioning did not yield persistent radiographic vertebral height maintenance, these fractures are best approached similarly to kyphoplasty for osteoporotic fractures, with aims to restore vertebral height (Cawley et al., 2011; Ng et al., 2016).

Most conventional transpedicular instrumentation designs feature top-loading screws which means that revising a screw requires open surgery with removal of a significant proportion if not all of the rod. On revision, the “halo” of osteolysis around the screw threads or porosity of bone may be too extensive to accommodate a larger or longer screw. Other anatomical techniques include changing the screw trajectory (very difficult) or extending the instrumentation to another vertebral level (typifying a longer rod-working length) (Fujibayashi et al., 2013). The defect thus mandates inserting a composite such as PMMA, hydroxyapatite granules, calcium phosphate, or bone graft to augment the pull-out strength. PMMA displays a greater pull-out strength than milled or matchstick bone (Pfeifer et al., 1994), with equivalence between fenestrated and solid pedicle screws (Leichtle et al., 2016). The operator must plan meticulously but also display the technical versatility to pursue additional options to achieve optimal screw stability.

The shape of the osteolytic “halo” has been described as ellipsoid at S1, greatest at the screw base and tapering off towards the screw tip (Kocak et al., 2013)^,^ as opposed to the PJK/PJF mechanism of failure. Thus, sufficient backfilling of the screw cavity is important to achieve stability at the screw base and a transpedicular approach was mentioned by one study for S1 (Yılmaz et al., 2022). Critically, if aiming for cement backfilling, evaluate pedicle integrity to prevent cement extrusion. Allowing sufficient pre-operative time promotes sclerosis of the “halo” margin to become impermeable to interdigitation of cement. This may explain a reduced rate of cement embolization in this series, as seen on de novo cannulated cement augmented screw insertion.

PMMA use in salvage cases is a recent phenomenon given the increasing prevalence of degenerative spinal deformity and greater levels of spinal fusion (Fujishiro et al., 2019). This series of mechanical complications encompasses heterogenous cases as a start point for discussion on this subject. Reporter bias was likely given the lack of external observers and subjective definition of instrumentation failure and follow-up was in general short, 3–24 months. The time period between initial surgery and salvage cement injection was thus not instructive (Table 1). Typically, failure of a spinal construct would become symptomatic within a year. Biomechanical studies are needed to compare this with other conventional techniques. Loose iliac screws have been assessed on cadaveric models post-pullout and then post-cementation demonstrating an effective stability effect after PMMA augmentation (Decker et al., 2019). However, its merits are the minimal invasiveness and morbidity yielding early pain relief, with an 15% incidence of conversion to open surgery (11/72 all-pathology patients). Questions remain regarding durability, potential to compromise further surgery and its application in the presence of infection. Patients who can tolerate more robust revision surgery may be more appropriately treated with a revision screw insertion and PMMA application (Kang et al., 2011). This study reports low-certainty of evidence, thus one may infer a similarly low strength of recommendation. In the absence of other data, it may be helpful for decision making in selected patients.

## 4 Conclusion

Developing a safe and feasible procedure that avoids high morbidity spinal surgery is a matter of great importance for healthcare providers, users and policy makers. While revision remains the gold standard, this systematic review identified that trialling percutaneous cementation for a failing screw-bone interface is worth consideration in selected cases, as an effective salvage procedure with minimal morbidity, appropriate for older or sicker patient and accepting that simply injecting PMMA does not address the underlying pathology. This rarely used technique is manifested by the low numbers of reported cases. This technique is within the remit of both the spine surgeon and interested interventional radiologists but is in its infancy and thus best performed within a multidisciplinary setting at a specialist center.

## Funding disclosures

The authors received no financial or material support for the research, authorship, and/or publication of this article.
